# Using clear plastic CD cases as low‐cost mini‐rhizotrons to phenotype root traits

**DOI:** 10.1002/aps3.11340

**Published:** 2020-04-19

**Authors:** Steven T. Cassidy, Audrey A. Burr, Rachel A. Reeb, Ana L. Melero Pardo, Kamron D. Woods, Corlett W. Wood

**Affiliations:** ^1^ Department of Biological Sciences University of Pittsburgh Pittsburgh Pennsylvania USA; ^2^ Present address: Department of Biology University of Florida Gainesville Florida USA

**Keywords:** *Medicago*, mini‐rhizotron, rhizobia, root phenotype, symbiosis

## Abstract

**Premise:**

We developed a novel low‐cost method to visually phenotype belowground structures in the plant rhizosphere. We devised the method introduced here to address the difficulties encountered growing plants in seed germination pouches for long‐term experiments and the high cost of other mini‐rhizotron alternatives.

**Methods and Results:**

The method described here took inspiration from homemade ant farms commonly used as an educational tool in elementary schools. Using compact disc (CD) cases, we developed mini‐rhizotrons for use in the field and laboratory using the burclover *Medicago lupulina*.

**Conclusions:**

Our method combines the benefits of pots and germination pouches. In CD mini‐rhizotrons, plants grew significantly larger than in germination pouches, and unlike pots, it is possible to measure roots without destructive sampling. Our protocol is a cheaper, widely available alternative to more destructive methods, which could facilitate the study of belowground phenotypes and processes by scientists with fewer resources.

Phenotyping root traits is a prerequisite for addressing fundamental basic and applied questions in plants, ranging from the evolutionary ecology of belowground species interactions (Wood et al., 2018) to how root functional traits influence crop yield (Hammond et al., 2009). The ecology and evolution of belowground mechanisms and species interactions represent an understudied area of plants due to the hidden nature of roots. Yet, despite the challenges associated with studying belowground processes in plants, there has been a recent surge of interest in these processes (see e.g., van de Voorde et al., 2012) due to their potential to answer important questions about crop productivity and species interactions (Vamerali et al., 2012). Biologists primarily use rhizotrons—chambers used to non‐invasively observe roots—to study belowground processes in living plants. Studies utilizing rhizotrons have shed light on root functions in plant communities across virtually all ecosystems (Hendrick and Pregitzer, 1996). Rhizotrons range in size from underground windowed facilities built under forests (Potvin and Lilleskov, 2017) to small plastic bags (i.e., seed germination pouches) used to study herbaceous plants in a laboratory setting (You et al., 2018). Researchers use rhizotrons to observe rhizobacteria‐associated phenotypes in legume roots including mutualist impacts (Sepúlveda‐Caamaño et al., 2018), nodulation formation and effectiveness (Yates et al., 2016), and parasitic plant resistance (Fernández‐Aparicio et al., 2012). Additional phenotypic applications include root ontogeny (Keng, 1988), environmental variation (Moser et al., 2010), and root architecture (Eberbach et al., 2013).

However, the dilemma in working with rhizotrons resides in their high price (Jeudy et al., 2016) and inadequate replication of the physical structure of natural soils (Mathieu et al., 2015). Many researchers build their own mini‐rhizotrons using plexiglass and plastic tubing (James et al., 1985), but this is time consuming, labor intensive, and impractical for large‐scale experiments. Most alternatives to rhizotrons—e.g., “shovelomics” (Trachsel et al., 2011), trench profiles (Nielsen et al., 1997), and soil cores (Benjamin and Nielsen, 2004)—require destructive sampling, reducing sample size with every harvest and precluding the acquisition of time series data. Few non‐destructive rhizotron alternatives exist; those that do (methods like X‐ray computed tomography) cost exorbitant amounts of money (Wu and Guo, 2014). A low‐cost method to visually assess the rhizosphere could integrate root phenotypes into the basic and applied research programs through which we understand other plant traits. In particular, developing a low‐cost mini‐rhizotron has the potential to democratize this subfield by lowering the barrier to entry for scientists, especially those working at institutions or in regions of the world with fewer resources. Low‐cost mini‐rhizotrons could also facilitate the development of root‐focused laboratory exercises for undergraduate and high school classrooms. Overall, the availability of inexpensive mini‐rhizotrons, available off‐the‐shelf with minimal modification from non‐specialist suppliers, could foster a more inclusive and diverse community of scientists working on belowground processes in plant systems.

Here we describe a cost‐efficient, scalable method to visualize whole root systems by growing plants in standard clear plastic compact disc (CD) jewel cases (Fig. 1). This version of the mini‐rhizotron allows biologists to phenotype the roots of plants through time while experiencing realistic soil conditions, without sacrificing sample size for time‐series data and at a fraction of the cost of most rhizotrons. Our method could prove useful for the study of plant interactions with nitrogen‐fixing bacteria (rhizobia), mycorrhizal fungi, parasites, and other elements of the root microbiome, especially those that form visible structures on the roots or otherwise change root morphology. We piloted this method in the field and lab in *Medicago lupulina* L., a legume that is native to Eurasia and naturalized throughout North America (Turkington and Cavers, 1979). *Medicago lupulina* is a member of the burclover genus *Medicago* L., which also contains *M. truncatula* Gaertn., a genetic model for belowground plant–microbe interactions, and *M. sativa* L. (alfalfa), an important agricultural crop.

**Figure 1:**
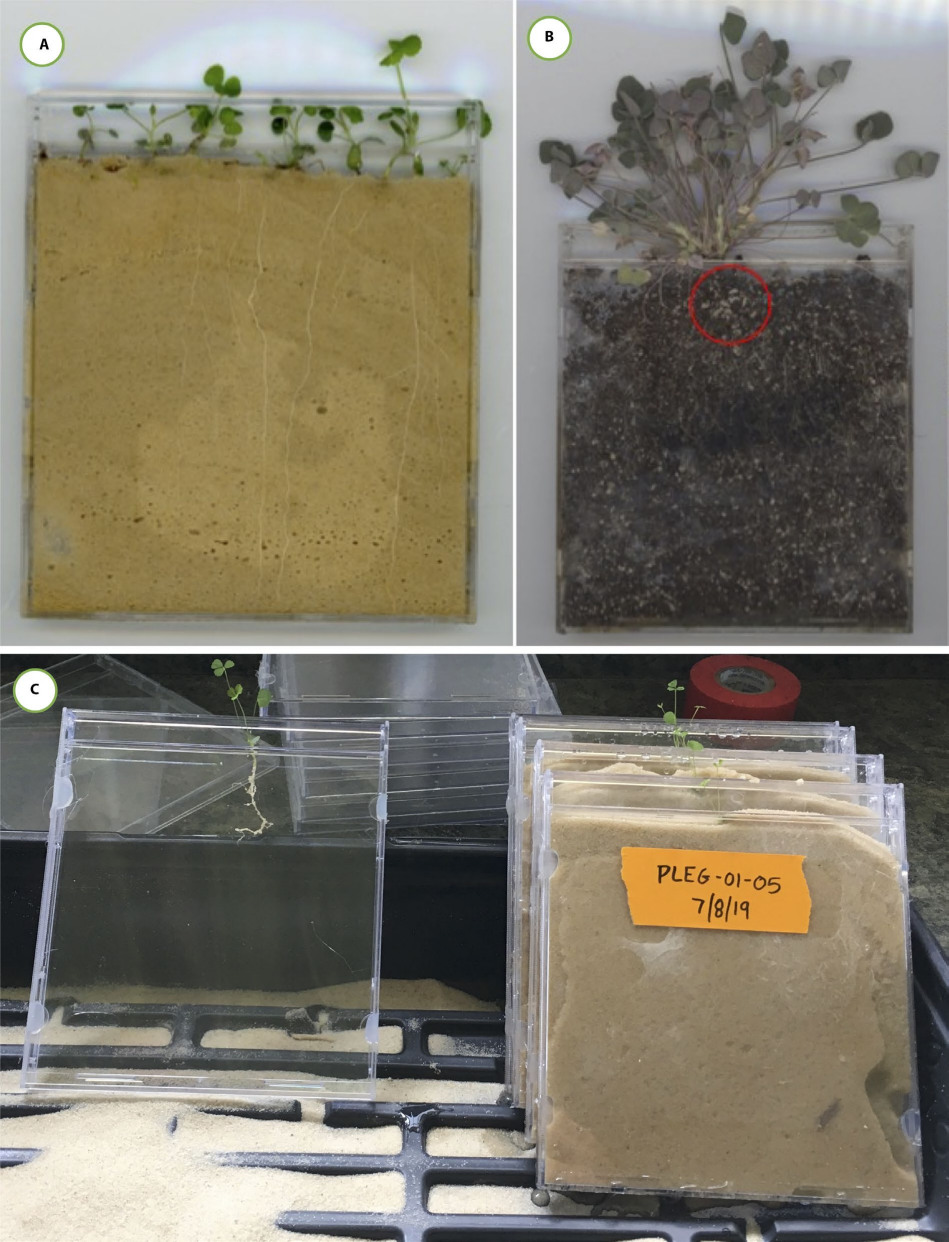
(A) Several young *Medicago lupulina* plants growing in CD mini‐rhizotrons with sand as the growing medium. (B) One older *M. lupulina* plant growing in soil containing perlite and vermiculite, proving the difficulties of phenotyping developing rhizobial nodules (circled in red). (C) Transplanting *M. lupulina* seedlings into CD mini‐rhizotrons, with sand as the growing medium, with the CD cases leaning against each other at an angle to force root growth against the back of the mini‐rhizotron.

## METHODS AND RESULTS

### CD mini‐rhizotron setup and protocol overview

We obtained standard‐sized, clear plastic CD jewel cases (14.2 cm × 12.5 cm × 1 cm) (Verbatim, Charlotte, North Carolina, USA) made of polystyrene from a retail office supply store, but a cheaper option is available from ULINE (model: S‐8111C; ULINE, Pleasant Prairie, Wisconsin, USA; https://www.uline.com/) at a cost of US$54 for a carton of 200 (US$0.27 per case; cost as of March 2020). For a detailed protocol describing mini‐rhizotron setup and plant growth, see Appendix 1; for root imaging see Appendix 2.

Briefly, the protocol consists of the following steps. First, we removed and recycled the black media trays (which hold CDs in place). These media trays come with the outer case from the retail supplier; ULINE offers an inexpensive option to purchase jewel cases without the tray. “Slimline” jewel cases (0.5‐cm diameter) cannot function as mini‐rhizotrons because the media tray is built into the back case and is not removable. Second, to prepare CD mini‐rhizotrons for planting, we plugged the sides of the CD case to prevent growth medium from spilling out. We tested three methods of sealing the CD cases (hot glue, duct tape, and silicone) and found that hot glue has the fastest application and drying time, has the strongest integrity, and is inexpensive relative to silicon alternatives. Third, we filled the CD case with sand as a growth medium (for other potential growth media; see Appendix 1). Fourth, we transplanted germinated seedlings (or planted seeds; see Appendix 1) into the CD mini‐rhizotrons (one seedling per CD mini‐rhizotron). Fifth, to shield the roots from light and prevent algal growth when grown in the lab, we enclosed each CD mini‐rhizotron in an opaque sleeve made from a sandwich‐size zip‐top plastic bag (16.5 cm × 14.9 cm) and duct tape (Appendix 1). Lastly, we grew plants in the CD mini‐rhizotrons leaning against one another at an angle in planting trays in the lab, and buried in sand at an angle in the field; this forces the plant roots to grow against the back of the case, which increases visibility from the outside of the case. We also grew plants without tilting the CD case, which is a preferred practice when using minirhizotrons (Vamerali et al., 2012), but found these root systems harder to observe. Tilting was also beneficial because, when the stem was upright, it grew against the plastic lip at the top of the CD case opening, which resulted in the shoot bending and getting stuck as it grew larger. Plants growing in mini‐rhizotrons can receive water from the bottom or top, by soaking CDs in water‐filled trays or misting with a hose, respectively. We preferred to bottom‐water mini‐rhizotrons and mist the top as needed to prevent the plants from drying out.

### Comparison with existing methods

#### Pots and seed germination pouches

We compared the growth and survival of *M. lupulina* plants grown in CD mini‐rhizotrons with two existing methods: Cone‐tainer pots (Stuewe & Sons Inc., Tangent, Oregon, USA) and CYG seed germination pouches (Mega International, Newport, Minnesota, USA). We scarified all *M. lupulina* seeds used in this study with a razor blade and sterilized them in bleach and ethanol. Seeds were germinated in the dark at 4°C for 72 h on sterile water agar plates and incubated at room temperature for 16 h prior to planting to induce radicle elongation (Garcia et al., 2006). We sterilized all materials prior to the start of the experiment with either a dilute bleach solution (CD rhizotrons and duct tape sleeves) or twice autoclaved at 121°C (germination pouches, Cone‐tainer pots, and sand).

We grew plants in CD mini‐rhizotrons (*N* = 20) and Cone‐tainers (*N* = 20) from seeds collected from five wild *M. lupulina* populations in Linesville, Pennsylvania (41.65°N, −80.42°W). We planted these seeds into 1.25‐inch‐deep plug trays and grew them in a greenhouse for one week at the University of Pittsburgh (16 : 8 h light : dark cycle; daytime temperature: 23°C; nighttime temperature: 19°C). We then transplanted the seedlings into their respective growing treatment—CD mini‐rhizotrons or Cone‐tainers—and allowed them to grow for six weeks. We grew all plants in sterilized sand to more easily see root phenotypes (Fig. 1A, B), and watered them as needed with deionized water.

Plants were grown in seed germination pouches (*N* = 81) from seeds collected from five wild populations of *M. lupulina*, collected from roadside populations in Giles and Craig counties in southwestern Virginia, near Mountain Lake Biological Station (37.37°N, −80.52°W). For germination pouches, we inoculated half of the plants with the mutualistic bacteria *Ensifer meliloti* (strain Em1022), a species of rhizobia that lives inside specialized root organs called nodules and fixes nitrogen for the host plant. We bundled germination pouches into groups of 15, wrapped aluminum foil around the roots, and placed them in sterilized, clear plastic boxes in a growth chamber (16 : 8 light : dark cycle; daytime temperature: 23°C; nighttime temperature: 19°C). Germination pouches received deionized water and 5–7 mL of nitrogen‐free Fahraeus fertilizer (Barker et al., 2006) as needed.

Plants in all treatments (CD mini‐rhizotrons, Cone‐tainer pots, and germination pouches) grew for six weeks prior to harvest. Plants grown in germination pouches were sourced from a different population than the Cone‐tainers and CD cases. However, given that a previous study did not detect differences in growth between *M. lupulina* populations spanning Delaware to Ontario, growth differences between populations are unlikely to account for the differences we observed between the CD mini‐rhizotrons, Cone‐tainers, and seed germination pouches (Harrison et al., 2017).

#### CD mini‐rhizotron performance in the field

To test the feasibility of CD mini‐rhizotrons to grow plants under ambient environmental conditions in a field setting, we grew *M. lupulina* plants in CD mini‐rhizotrons (*N* = 20) and Cone‐tainers (*N* = 20) under common garden conditions at the Pymatuning Laboratory of Ecology in Pennsylvania (41.65°N, −80.42°W). This site is an old‐field ecosystem historically used for agriculture and currently maintained for experimental use. Background vegetation removal occurred by soil tilling, and we erected a 2.5‐m‐tall fence to prevent deer and other large herbivores from accessing the plants. We planted CD mini‐rhizotrons and Cone‐tainers into large plastic storage bins filled with sand and gravel, which we sunk into the ground to buffer changes in moisture and temperature. We inoculated all plants with rhizobia (*Ensifer* spp.), which we cultured from *M. lupulina* plants growing adjacent to our field site. We covered plants with shade cloth to prevent UV shock and replaced dead plants for the first week after initial deployment. Plants grown in the field experienced ambient rainfall with supplements of well water as needed. We replaced 10% of our field plants and none of our growth chamber plants grown in CD cases, so future studies that use this method in the field should germinate about 10% more plants to account for initial mortality.

We imaged CD mini‐rhizotrons on a flatbed scanner (Appendix 2); root phenotyping can occur with these images in conjunction with existing software (e.g., ImageJ; Carotenuto et al., 2019). However, imaging the roots on a flatbed scanner is not required for root morphological measurements. Root measurements can be done by visually assessing with the naked eye. For example, root colonization of nodules could be counted, and root lengths could be obtained by tracing roots with string and measuring string with measuring tape. To compare CD mini‐rhizotrons to existing growing methods (Cone‐tainer pots and germination pouches), we measured plant survival, shoot height, and root length. We measured the length of the longest root to the nearest millimeter on a ruler after plants were harvested to compare root size between growing methods. We also measured root length in situ in CD mini‐rhizotrons by tracing the longest visible root with a length of string and then measuring the string. To test whether in situ measurements in the CD mini‐rhizotrons accurately reflect true root length, we measured the correlation between post‐harvest root length and the pre‐harvest in situ measurements.

We used general linear models (“lm” function) and Tukey post‐hoc tests in the statistical programming language R (R Core Team, 2018) executed in the emmeans package (Lenth et al., 2019) to compare stem height across the three growing methods and performed all analyses in R version 3.5.1. In these models, stem height and log‐transformed root length were our response variables, and growing method was our fixed effect predictor. Our models assumed a Gaussian error distribution. We log‐transformed root length to conform to regression assumptions (normality of residual variation, homogeneity of variance), which we tested for all models using the DHARMa package in R. We used a chi‐square test to compare survival across the three growth methods.

### Comparison results

**Figure 2:**
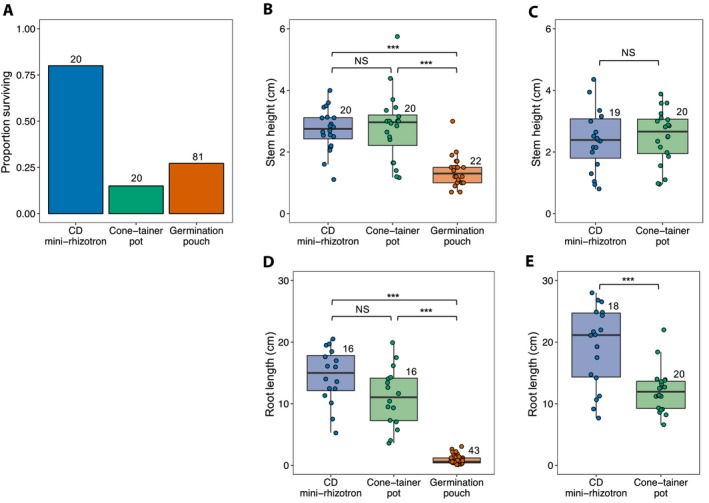
*Medicago* *lupulina* survival (A), stem height (B, D), and root length (C, E) when grown in CD mini‐rhizotrons, Cone‐tainer pots, and germination pouches for plants grown in growth chambers (A, B, D) and in a field common garden (C, E). Sample sizes are indicated above each bar (A) or box (B–E). *P < 0.05, **P < 0.01, ***P < 0.001, NS = not significant.

Plants grown in CD mini‐rhizotrons had a higher survival rate than those grown in Cone‐tainer pots or germination pouches (*χ* ^*2*^ = 23.80, *P* < 0.001) (Fig. 2A). Under controlled growth chamber conditions, stem height and root length differed among growing methods (growth chamber: *F*_2,59_ = 21.65, *P* < 0.001; root length: *F*_2,72_ = 169.35, *P* < 0.001), a pattern driven by slower growth in the germination pouches (Fig. 2B). Plants grew taller in CD mini‐rhizotrons than in germination pouches (*t*‐ratio = 5.48, *df* = 59, *P* < 0.001). Stem height did not differ significantly between CD mini‐rhizotrons and Cone‐tainer pots in either the growth chamber (*t*‐ratio = −0.332, *df* = 59, *P* = 0.914) or in the field common garden (*F*_1,37_ = 0.142, *P* = 0.701) (Fig. 2B, C). Roots grew longer in CD mini‐rhizotrons than in growth pouches (*t*‐ratio = 15.457, *df* = 72, *P* < 0.001), but not Cone‐tainer pots (growth chamber: *t*‐ratio = 1.368, *df* = 72, *P* = 0.36, field: *F*_1,36_ = 15.057, *P* < 0.001) (Fig. 2D, E). Aboveground growth in CD mini‐rhizotrons did not differ significantly from growth in pots in either the growth chamber (*t*‐ratio = −0.332, *df* = 59, *P* = 0.914) or in the field common garden (*F*_1,37_ = 0.142, *P* = 0.701) (Fig. 2B, C). Finally, we found a strong correlation between pre‐ and post‐harvest root measurements (*r* = 0.76, *t*‐ratio = 4.395, *df* = 14, *P* < 0.001), indicating that in situ root measurements in CD mini‐rhizotrons accurately capture root system size. We observed very little nodulation with rhizobial bacteria in any of our three treatments, so we could not present data on the rhizobial mutualism here.

### Method comparisons

CD cases are a novel, cheap, and reusable alternative to the traditional mini‐rhizotron and combine the benefits of both Cone‐tainers and growth pouches. Researchers use Cone‐tainers as a standard low‐cost growing method for plants grown in high densities, but root phenotype assessments require sacrifices in sample size. In our study, plants grown in the CD cases grew just as large aboveground and larger belowground in both the lab and the field (Fig. 2). We argue that our results indicate CD mini‐rhizotrons are actually better than Cone‐tainers for root measurements because plants grow equally large shoots in both growing methods, but CD mini‐rhizotrons facilitate growth of longer roots (Fig. 2).

In germination pouches, root phenotypes can be tracked through time and imaged at high contrast, but the pouches do not represent natural or favorable conditions for plant growth. In our study, plants in germination pouches grew much shorter roots relative to plants grown using the other methods (Fig. 2), which makes root phenotyping challenging. In addition, persistent fungal growth on and around the roots of *M. lupulina* plants grown in germination pouches appeared to limit the growth and survival of the plants. This led to drastically smaller plants with a greatly decreased survival rate (Fig. 2), which demonstrates the utility of using CD mini‐rhizotrons as an alternative. Maintaining consistent moisture levels within the pouches also proved difficult and time consuming. Even with daily monitoring, the growth pouches would experience different rates of moisture loss based on their position within a bundle and within the growth chamber. CD mini‐rhizotrons provide the collective benefit of tracking root phenotypes through time, while providing ample space and growth medium for plants to realize their growth potential.

## CONCLUSIONS

The abundance and diversity of root phenotyping methods is clear (James et al., 1985; Nielsen et al., 1997; Benjamin and Nielsen, 2004; Trachsel et al., 2011; Atamian et al., 2012; Wu and Guo, 2014; Mathieu et al., 2015; Jeudy et al., 2016; Potvin and Lilleskov, 2017), but the labor intensity and cost‐limitation of effective rhizotrons leave vital root measurements inaccessible to some plant biologists. The CD mini‐rhizotrons described here offer a cost‐effective and simple method for researchers to perform time‐series experiments and incorporate large sample sizes into their experimental design. Our method does have a few limitations. Due to the small size of CD mini‐rhizotrons, this protocol is best applied to young plants and small herbaceous species. Rhizotrons may hide root phenotype measurements within the center of the growing medium, especially for plants with bulky root structures, but this problem is minimized by the thin nature of the CD case. Nonetheless, CD mini‐rhizotrons provide researchers the opportunity to phenotype root traits and the potential to study root‐associated symbioses, such as rhizobacteria and arbuscular mycorrhizal fungi, at a relatively low cost. This method democratizes new research opportunities in the root microbiome for agriculture, community ecology, and evolutionary biology, particularly for researchers with limited resources.

## Figures and Tables

**Figure A1:**
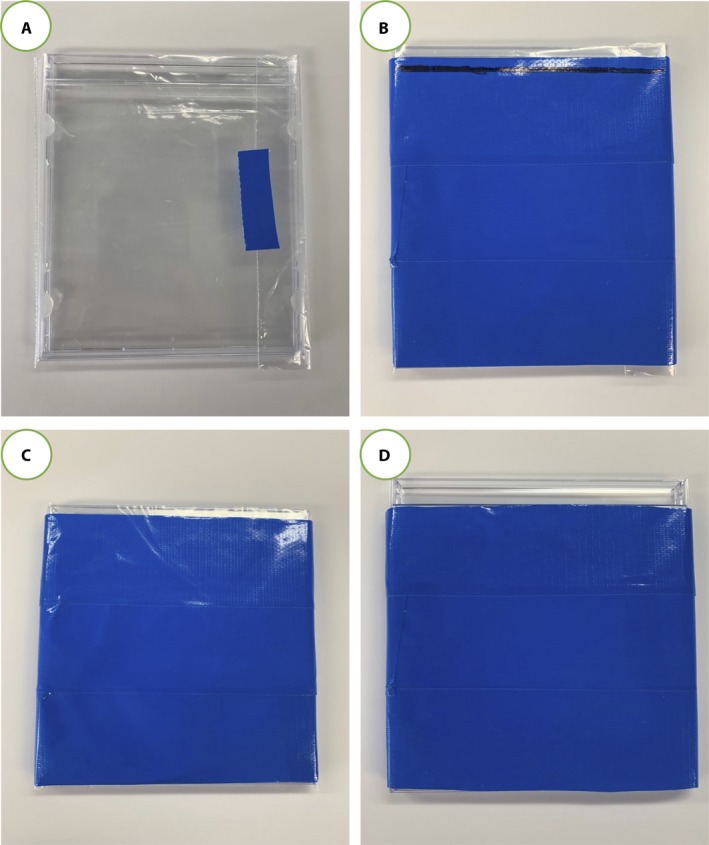
Steps to make CD case sleeves. (A) Cut the top off a zip‐top sandwich bag and secure around the CD case with tape. (B, C) Wrap duct tape around the CD case, covering the entire bag, and draw a line on the sleeve at the bottom of the spine slot. (D) Trim the sleeve along the line.

## Data Availability

The data and associated R scripts are available through the open access repository Figshare (data: https://doi.org/10.6084/m9.figshare.12021075; analysis: https://doi.org/10.6084/m9.figshare.12021084).

## APPENDIX 1 Detailed protocol for CD mini‐rhizotron setup and plant growth.

### Preparing the CD case

1. Remove media tray from CD case.
2. Close CD case and fill any large holes with hot glue, except the slot where the spine is. Avoid forming large bumps (the hot glue can be smoothed with a razor blade before it dries if needed).

[Note: Hot glue has the fastest application and drying time and is inexpensive compared to silicon. Hot glue also is highly durable relative to duct tape.]

### Making CD case sleeves

1. Cut the zip‐lock off the top of a zip‐top sandwich bag (16.5 cm × 14.9 cm).
2. Place a CD case inside and fold the edge of the bag over, so that it fits the CD case but is not tight, and secure the fold with a small piece of duct tape (Fig. A1A).
3. Wrap the duct tape evenly around the CD case, covering the entire bag; however, be sure not to tape the bag tightly against the CD case (Fig. A1B).
4. Use a permanent marker to draw a line on the sleeve at the bottom of the spine slot (Fig. A1C).
5. Remove the sleeve and trim the CD case along the line.
6. Trim the bottom of the sleeve off so that it becomes a sleeve (Fig. A1D).

### Filling the CD case

1. Fill a container, such as an autoclave bin or watering tray, with approximately one‐half inch of water.
2. Using a weigh boat as a scoop, pour dry growth medium (e.g., sand, soil) into the top spine slot to fill the CD case to the brim.
3. Stand the CD case upright in the water to allow the water to travel up the CD case until all the sand is damp.
  - If the sand runs out of the CD case, refill the CD case to the brim and dampen the sand using a beaker or squirt bottle.
4. Remove the CD case from the water and dry the outside with a paper towel.
5. Put the sleeve over the CD case.
  - If the sleeve is too tight, put your fingers inside the sleeve and gently pull outward, until it fits over the CD case.

[Note: We piloted the use of agar as a growth medium in our CD cases, but it dissolved in water and washed away in a couple days during watering. Sand and soil both proved viable options for growing plants. Avoid soil with too much perlite and vermiculite, as both proved difficult for root phenotyping purposes.]

### Planting seedlings

1 Follow an appropriate germination protocol for your study species.
  [Note: This protocol is suitable for small plants whose root systems will not be constrained by the size of the CD case.]
2 Age of seedling:
  - Newly germinated: Push forceps into the substrate to make a hole. (Make the hole deep enough to fit the radicle straight into the soil).
  - Older seedling: Pour the substrate around the roots, taking care that the aboveground biomass does not get buried (Fig. 1C).
3 Plant the radicle down into the substrate.
4 Use a spray or squirt bottle to moisten the substrate.

[Note: Germinating seeds in the CD mini‐rhizotrons was not more time efficient for *Medicago lupulina*. It was still necessary to manipulate the seeds so that their radicles pointed downward; despite the orientation, we germinated them in the mini‐rhizotrons.]

### Plant care

1. This section depends on the individual plants used. Plants should be watered as needed.
2. Allow for watering trays to dry out for a period of time (12–24 h) to prevent root rot.
3. To prevent drying out, do not leave plants drying for more than 48 h.

### Sterilizing and re‐using CD cases

1. Soak CD cases and CD case sleeves in a bleach solution (the solution is sufficiently concentrated when the bleach scent is detectable) for ~15–20 min.
2. Rinse with water until the bleach scent is no longer detectable.

[Note: Your chosen substrate may be autoclaved prior to filling the CD cases.]

## APPENDIX 2 Detailed protocol for root imaging with the CD mini‐rhizotron system.

### Adjusting your scanner settings

1. Turn on the scanner (Epson Perfection V600 Photo Scanner, Epson, Long Beach, California, USA; US$299.99) and open up the scanner application on your computer.
2. Go to professional mode.
3. Set the resolution to 600 ppi.
4. Choose to set the file format to save as a TIF file.
5. Increase the saturation and contrast to 15.
6. Choose the location you want your scanned file to save to.

[Note: To determine the resolution needed for your purpose, scan a sample CD at different resolutions and record the time it takes to a complete a single scan. Multiply that number by 2 and then by your sample size to determine how long it will take to complete a scan of all individuals. This allows you to determine appropriate resolution for your purposes. Saturation and contrast may also need to be adjusted according to your specific scanner's settings.]

### Scanning the CD case

1. Cut projector film to protect the scanner bed and top scanner panel.
2. Tape the projector film over the scanner bed and top panel.
3. Use ethanol to sanitize the projector film for sterile conditions if needed.
4. Remove the CD case sleeve and lay the CD case flat on the scanner bed.
5. Close the scanner and scan the CD case using the “scan” option on your computer.
6. Name the file accordingly. Be sure to identify in the file name whether the front or back of the case was scanned.
7. Flip the CD case over and scan again, repeating steps 4 and 5.
8. Put CD case sleeve back on.

### Software for image analysis

The scanned images of CD mini‐rhizotrons can be imported into existing software for root analysis, such as SmartRoot (Lobet et al., 2011), BRAT (Slovak et al., 2014), and Fiji (Schindelin et al., 2012). These tools are free to download and platform independent. Additional software is also available online at the Plant Image Analysis portal (Lobet et al., 2013; https://www.quantitative-plant.org/), a database for plant image analysis.

## References

[aps311340-bib-0001] Atamian, H. S. , P. A. Roberts , and I. Kaloshian . 2012 High and low throughput screens with root‐knot nematodes *Meloidogyne* spp. JoVE (Journal of Visualized Experiments) 61: e3629. 10.3791/3629 PMC3402051 22434014

[aps311340-bib-0002] Barker, D. G. , T. Pfaff , D. Moreau , E. Groves , S. Ruffel , M. Lepetit , S. Whitehand , et al. 2006 Growing *M. truncatula*: Choice of substrates and growth conditions, 26 *In* Mathesius U., Journet E., and Sumner L. [eds.], *Medicago truncatula* handbook. Noble Research Institute, Ardmore, Oklahoma, USA.

[aps311340-bib-0003] Benjamin, J. G. , and D. C. Nielsen . 2004 A method to separate plant roots from soil and analyze root surface area. Plant and Soil 267: 225–234.

[aps311340-bib-0004] Carotenuto, G. , I. Sciascia , L. Oddi , V. Volpe , and A. Genre . 2019 Size matters: Three methods for estimating nuclear size in mycorrhizal roots of *Medicago truncatula* by image analysis. BMC Plant Biology 19: 180. 31054574 10.1186/s12870-019-1791-1 PMC6500585

[aps311340-bib-0005] Eberbach, P. L. , J. Hoffmann , S. J. Moroni , L. J. Wade , and L. A. Weston . 2013 Rhizo‐lysimetry: Facilities for the simultaneous study of root behaviour and resource use by agricultural crop and pasture systems. Plant Methods 9: 3. 23363534 10.1186/1746-4811-9-3 PMC3579728

[aps311340-bib-0006] Fernández‐Aparicio, M. , A. Moral , M. Kharrat , and D. Rubiales . 2012 Resistance against broomrapes (*Orobanche* and *Phelipanche* spp.) in faba bean (*Vicia faba*) based in low induction of broomrape seed germination. Euphytica 186: 897–905.

[aps311340-bib-0007] Garcia J. , D. G. Barker , and E.‐P. Journet . 2006 Seed storage and germination *In* Mathesius U., Journet E., and Sumner L. [eds.], *Medicago truncatula* handbook. Noble Research Institute, Ardmore, Oklahoma, USA Website http://www.noble.org/MedicagoHandbook [accessed 13 April 2020].

[aps311340-bib-0008] Hammond, J. P. , M. R. Broadley , P. J. White , G. J. King , H. C. Bowen , R. Hayden , M. C. Meacham , et al. 2009 Shoot yield drives phosphorus use efficiency in *Brassica oleracea* and correlates with root architecture traits. Journal of Experimental Botany 60: 1953–1968. 19346243 10.1093/jxb/erp083

[aps311340-bib-0009] Harrison, T. L. , C. W. Wood , I. L. Borges , and J. R. Stinchcombe . 2017 No evidence for adaptation to local rhizobial mutualists in the legume *Medicago lupulina* . Ecology and Evolution 7: 4367–4376. 28649348 10.1002/ece3.3012 PMC5478075

[aps311340-bib-0010] Hendrick, R. L. , and K. S. Pregitzer . 1996 Applications of minirhizotrons to understand root function in forests and other natural ecosystems. Plant and Soil 185: 293–304.

[aps311340-bib-0011] James, B. R. , R. J. Bartlett , and J. F. Amadon . 1985 A root observation and sampling chamber (rhizotron) for pot studies. Plant and Soil 85: 291–293.

[aps311340-bib-0012] Jeudy, C. , M. Adrian , C. Baussard , C. Bernard , E. Bernaud , V. Bourion , H. Busset , et al. 2016 RhizoTubes as a new tool for high throughput imaging of plant root development and architecture: Test, comparison with pot grown plants and validation. Plant Methods 12: 31. 27279895 10.1186/s13007-016-0131-9 PMC4897935

[aps311340-bib-0013] Keng, J. 1988 Monitoring corn root development with a mini‐rhizotron in confined growth boxes. Agronomy Journal 80: 287–291.

[aps311340-bib-0014] Lenth, R. , H. Singmann , J. Love , P. Buerkner , and M. Herve . 2019 emmeans: Estimated marginal means, aka least‐squares means. R package version 1.2.3. Website https://CRAN.R-project.org/package=emmeans [accessed 17 March 2020].

[aps311340-bib-0015] Lobet, G. , L. Pagès , and X. Draye . 2011 A novel image‐analysis toolbox enabling quantitative analysis of root system architecture. Plant Physiology 157: 29–39. 21771915 10.1104/pp.111.179895 PMC3165877

[aps311340-bib-0016] Lobet, G. , X. Draye , and C. Périlleux . 2013 An online database for plant image analysis software tools. Plant Methods 9: 38. 24107223 10.1186/1746-4811-9-38 PMC3853381

[aps311340-bib-0017] Mathieu, L. , G. Lobet , P. Tocquin , and C. Périlleux . 2015 “Rhizoponics”: A novel hydroponic rhizotron for root system analyses on mature *Arabidopsis thaliana* plants. Plant Methods 11: 3. 25657812 10.1186/s13007-015-0046-x PMC4318444

[aps311340-bib-0018] Moser, G. , C. Leuschner , M. Roederstein , S. Graefe , N. Soethe , and D. Hertel . 2010 Biomass and productivity of fine and coarse roots in five tropical mountain forests stands along an altitudinal transect in southern Ecuador. Plant Ecology & Diversity 3: 151–164.

[aps311340-bib-0019] Nielsen, K. L. , J. P. Lynch , and H. N. Weiss . 1997 Fractal geometry of bean root systems: Correlations between spatial and fractal dimension. American Journal of Botany 84: 26–33. 11539495

[aps311340-bib-0020] Potvin, L. R. , and E. A. Lilleskov . 2017 Introduced earthworm species exhibited unique patterns of seasonal activity and vertical distribution, and *Lumbricus terrestris* burrows remained usable for at least 7 years in hardwood and pine stands. Biology and Fertility of Soils 53: 187–198.

[aps311340-bib-0021] R Core Team . 2018 R: A language and environment for statistical computing. R Foundation for Statistical Computing Vienna, Austria Website https://www.R-project.org [accessed 21 October 2019].

[aps311340-bib-0022] Schindelin, J. , I. Arganda‐Carreras , E. Frise , V. Kaynig , M. Longair , T. Pietzsch , S. Preibisch , et al. 2012 Fiji: An open‐source platform for biological‐image analysis. Nature Methods 9: 676–682. 22743772 10.1038/nmeth.2019 PMC3855844

[aps311340-bib-0023] Sepúlveda‐Caamaño, M. , M. Gerding , M. Vargas , E. Moya‐Elizondo , P. Oyarzua , and J. Campos . 2018 Lentil (*Lens culinaris* L.) growth promoting rhizobacteria and their effect on nodulation in coinoculation with rhizobia. Archives of Agronomy and Soil Science 64: 244–256.

[aps311340-bib-0024] Slovak, R. , C. Göschl , X. Su , K. Shimotani , T. Shiina , and W. Busch . 2014 A scalable open‐source pipeline for large‐scale root phenotyping of *Arabidopsis* . Plant Cell 26: 2390–2403. 24920330 10.1105/tpc.114.124032 PMC4114940

[aps311340-bib-0025] Trachsel, S. , S. M. Kaeppler , K. M. Brown , and J. P. Lynch . 2011 Shovelomics: High throughput phenotyping of maize (*Zea mays* L.) root architecture in the field. Plant and Soil 341: 75–87.

[aps311340-bib-0026] Turkington, R. , and P. B. Cavers . 1979 The biology of Canadian weeds. 33. *Medicago lupulina* L. Canadian Journal of Plant Sciences 59: 99–110.

[aps311340-bib-0027] Vamerali, T. , M. Bandiera , and G. Mosca . 2012 Minirhizotrons in modern root studies *In* Mancuso S. [ed.], Measuring roots: An updated approach, 341–361. Springer, Berlin, Germany.

[aps311340-bib-0028] van de Voorde, T. F. J. , W. H. van der Putten , and T. M. Bezemer . 2012 Soil inoculation method determines the strength of plant–soil interactions. Soil Biology and Biochemistry 55: 1–6.

[aps311340-bib-0029] Wood, C. W. , B. L. Pilkington , P. Vaidya , C. Biel , and J. R. Stinchcombe . 2018 Genetic conflict with a parasitic nematode disrupts the legume–rhizobia mutualism. Evolution Letters 2: 233–245. 30283679 10.1002/evl3.51 PMC6121810

[aps311340-bib-0030] Wu, J. , and Y. Guo . 2014 An integrated method for quantifying root architecture of field‐grown maize. Annals of Botany 114: 841–851. 24532646 10.1093/aob/mcu009 PMC4156117

[aps311340-bib-0032] Yates R. J. , J. G. Howieson , M. Hungria , A. Bala , G. W. O’Hara , and J. Terpolilli . 2016 Authentication of rhizobia and assessment of the legume symbiosis in controlled plant growth systems *In* Howieson J.G., and Dilworth M.J. [eds.],. Working with rhizobia, 73–108. Australian Centre for International Agricultural Research, Canberra, Australia.

[aps311340-bib-0031] You, J. , Y. Hu , and C. Wang . 2018 Application of seed germination pouch for culture and initial resistance screening of the soybean cyst nematode *Heterodera glycines* . Nematology 20: 905–909.

